# The interaction between strigolactones and other plant hormones in the regulation of plant development

**DOI:** 10.3389/fpls.2013.00199

**Published:** 2013-06-17

**Authors:** Xi Cheng, Carolien Ruyter-Spira, Harro Bouwmeester

**Affiliations:** Laboratory of Plant Physiology, Wageningen UniversityWageningen, Netherlands

**Keywords:** strigolactone, auxin, cytokinin, ethylene, gibberellins, hormone crosstalk, root and shoot architecture, phenotypic plasticity

## Abstract

Plant hormones are small molecules derived from various metabolic pathways and are important regulators of plant development. The most recently discovered phytohormone class comprises the carotenoid-derived strigolactones (SLs). For a long time these compounds were only known to be secreted into the rhizosphere where they act as signaling compounds, but now we know they are also active as endogenous plant hormones and they have been in the spotlight ever since. The initial discovery that SLs are involved in the inhibition of axillary bud outgrowth, initiated a multitude of other studies showing that SLs also play a role in defining root architecture, secondary growth, hypocotyl elongation, and seed germination, mostly in interaction with other hormones. Their coordinated action enables the plant to respond in an appropriate manner to environmental factors such as temperature, shading, day length, and nutrient availability. Here, we will review the current knowledge on the crosstalk between SLs and other plant hormones—such as auxin, cytokinin, abscisic acid (ABA), ethylene (ET), and gibberellins (GA)—during different physiological processes. We will furthermore take a bird's eye view of how this hormonal crosstalk enables plants to respond to their ever changing environments.

## Introduction

Plant hormones are small molecules derived from various essential metabolic pathways. They play critical roles during all developmental stages in plants, from early embryogenesis to senescence. Research on plant hormones started as early as the beginning of the last century and has resulted in the discovery of auxins, ethylene (ET), cytokinins (CK), gibberellins (GA), abscisic acid (ABA), brassinosteroids (BRs), jasmonic acid (JA), salicylic acid (SA), and the recently identified strigolactones (SLs). The biosynthetic pathways of these plant hormones have been mostly elucidated, with some minor exceptions, such as some missing steps in SL biosynthesis. Generally, plant hormones exert their effect locally at or near the site of biosynthesis or are mobile between different tissues. The mechanisms of hormone crosstalk can be diverse. Hormone signaling pathways are known to interact at the level of gene expression. A common crosstalk strategy is to control specific key components of signaling pathways of other hormones (Santner et al., 2009; Santner and Estelle, 2009). In this way, hormones might regulate synthesis (hormone levels), sensitivity (hormone response), and transport (hormone distributions) of other hormones.

During the last decade we have witnessed remarkable breakthroughs in plant hormone research, especially with the discovery of the SLs. With this discovery, plant scientists not only got a new tool to study hormonal regulation of plant development but were also triggered to critically assess existing hypotheses on hormone crosstalk mechanisms. SLs were known as host-derived germination stimulants for root parasitic plants such as the witchweeds (*Striga spp*.) and broomrapes (*Orobanche* and *Phelipanche spp*.) since the sixties of last century (Bouwmeester et al., 2003). Their function, as allelochemicals in symbiosis with arbuscular mycorrhizal (AM) fungi, was discovered only recently (Akiyama et al., 2005). SLs promote the establishment of mycorrhizal symbiosis which mainly facilitates the phosphate acquisition from the soil. Later, SLs were found to play a key role in shoot branching inhibition and thus were identified as a new group of plant hormones (Gomez-Roldan et al., 2008; Umehara et al., 2008). Their biological functions were further explored and it was discovered that they also exert their effects on different developmental processes including root development, seed germination, hypocotyl elongation, and secondary growth. Their conserved functions between different plant species are indicative of their indispensability in regulating plant development.

This review will focus on the current knowledge on the SLs and their hormonal crosstalk with other plant hormones such as auxin, CK, ABA, ET, and GA during bud outgrowth, root development, secondary growth, and seeds germination. We will furthermore take a bird's eye view of how this hormonal crosstalk enables the plant to respond to its ever changing environment, including shade and nutrient deprivation.

## SL biosynthesis and perception

**Figure 1:**
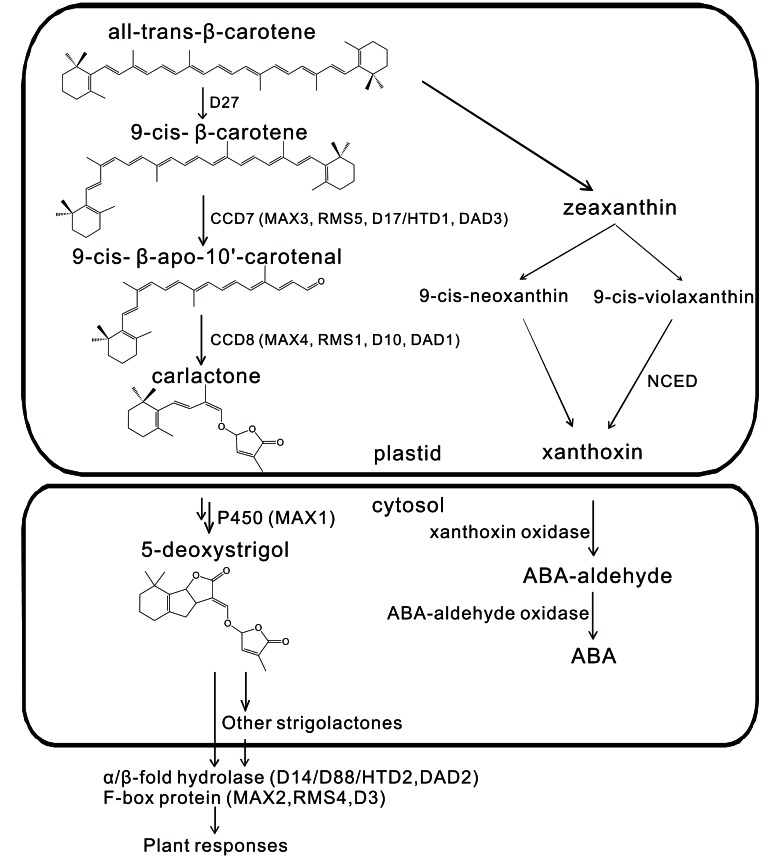
**Strigolactone and ABA biosynthetic pathways share a common origin at β-carotene**. Adapted and modified from Ruyter-Spira et al. (2013).

So far, at least 15 SLs have been structurally identified. They are typically composed of four rings (A–D). The A and B rings vary due to different side groups, while the C and D rings are highly conserved and seem to play an essential role in biological activity (Xie et al., 2010). Like ABA, SLs are also derived from the carotenoid pathway from which they are hypothesized to diverge at β-carotene (Matusova et al., 2005; Lopez-Raez et al., 2008; Rani et al., 2008) (see Figure 1). Interestingly, especially considering their common biosynthetic origin, a correlation between ABA levels and SLs production was observed in the ABA mutants *notabilis*, *sitiens*, and *flacca* and in plants treated with AbaminSG, an inhibitor of the ABA biosynthetic enzyme 9-*cis*-epoxycarotenoid dioxygenase (NCED). It was suggested that ABA may regulate SL biosynthesis (Lopez-Raez et al., 2010).

Several mutants with increased shoot branching phenotype have been identified in several plant species, including *more axillary growth* (*max*) in Arabidopsis (*Arabidopsis thaliana*), *ramosus* (*rms*) in pea (*Pisum sativum*), *dwarf* (*d*) or *high-tillering* *dwarf* (*htd*) in rice (*Oryza sativa*), and *decreased apical dominance* (*dad*) in petunia (*Petunia hybrida*). All these mutants are defective in SL biosynthesis or signaling. They form the basis for the discovery of genes involved in the SL biosynthetic and downstream signaling pathways. Key catalytic enzymes in the SL biosynthetic pathway include DWARF27 (D27) (Lin et al., 2009; Waters et al., 2012a), CAROTENOID CLEAVAGE DIOXYGENASE 7 and 8 (CCD7 and CCD8), and MAX1 (Booker et al., 2005; Kohlen et al., 2011) (see Figure 1). CCD7 and CCD8 are, respectively, encoded by the genes *MAX3*/*RMS5*/*D17(HTD1)*/*DAD3* (Morris et al., 2001; Booker et al., 2004; Zou et al., 2006; Drummond et al., 2009) and *MAX4*/*RMS1*/*D10*/*DAD1* (Foo et al., 2001; Sorefan et al., 2003; Snowden et al., 2005; Arite et al., 2007). Both the F-box protein MAX2/RMS4/D3 (Stirnberg et al., 2007; Yoshida et al., 2012) and the α/β-fold hydrolase D14/D88/HTD2/DAD2 (Arite et al., 2009; Liu et al., 2009; Gaiji et al., 2012; Hamiaux et al., 2012) have been shown to be involved in SL downstream signaling. More aspects about SLs biosynthesis, perception, and signaling as well as structure-function relationships have been nicely addressed and updated in several recent reviews (Janssen and Snowden, 2012; Ruyter-Spira et al., 2013; Zwanenburg and Pospisil, 2013).

## Interactions between auxin, SL, and cytokinin in the control of bud outgrowth

**Figure 2:**
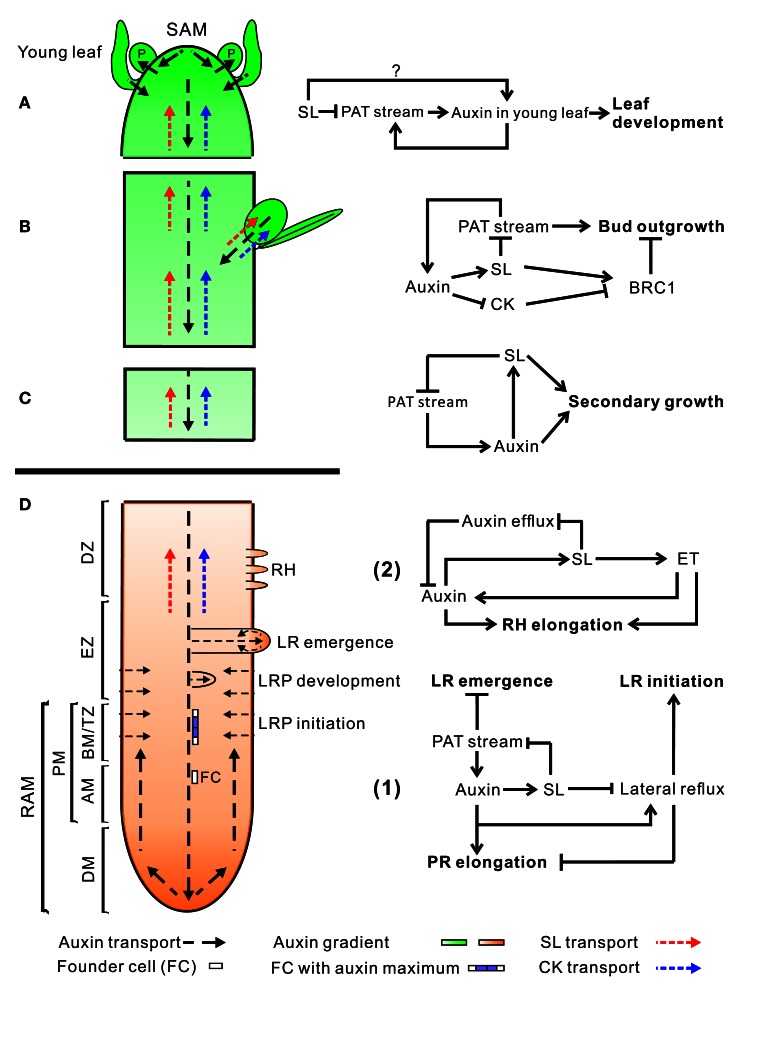
**An overview of auxin, SL, and CK transport within the plant (left) and hormone interactions during the regulation of shoot and root development (right)**. Auxin, strigolactone (SL), and cytokinin (CK) transport are represented by black, red, and blue dotted line, respectively. For hormone interactions (right), arrows represent promotion, while flat-ended lines indicate inhibition. **(A)** Auxin, produced in the shoot apical meristem (SAM) and young leaves, is transported basipetally through the stem in the polar auxin transport (PAT) stream toward the root apical meristem (RAM). Here, but probably also throughout the entire vasculature of the plant, it positively regulates SL biosynthesis (Hayward et al., 2009). As shown by GR24 feeding experiments, SLs transported through the xylem from the root to the shoot down-regulate the free auxin level in young leafs in a MAX2-dependent manner hereby controlling their development (Ruyter-Spira et al., 2011). SLs in the vasculature negatively affect PAT capacity (Crawford et al., 2010), as observed for NPA (Ljung et al., 2001), which negatively feeds back on auxin levels at the sites of biosynthesis. This long distance SL-auxin feedback mechanism, affects plant developmental processes as described below. **(B)** During the regulation of bud outgrowth, SLs reduce the capacity of the PAT stream in the main stem, leading to enhanced competition between buds to release their auxin into the stem (Crawford et al., 2010; Shinohara et al., 2013). On the other hand, SLs and CK are transported acropetally through the xylem and act directly in the buds to control their outgrowth through the joint regulation of TCP transcription factor BRC1 (Braun et al., 2012; Dun et al., 2012). **(C)** SLs have a direct positive effect on secondary growth by activating cell division in the vascular cambium in which they act downstream of auxin. The fact that the *max1* mutant still displays some residual cambium activity might point to a SL independent response to auxin. However, this remaining activity could also be due to residual SLs in these mutants (Agusti et al., 2011). **(D)** Hormone interactions during primary root (PR) elongation, lateral root (LR) initiation and development (1) and root hair (RH) elongation (2). (1) Auxin imported from the main PAT stream into the root stimulates SL production. SL export into the xylem and down regulation of the PAT stream feedback on auxin levels in the shoot as described under **(A)**. SL biosynthesis genes are specifically expressed in vascular tissue and the cortex of the proximal meristem of the root, through which the lateral auxin reflux toward the main PAT stream takes place. Therefore it is likely that locally synthesized SLs are controlling the efficiency of this reflux. Primary root elongation and lateral root initiation are determined by the auxin gradient inside the root tip, which is determined by auxin levels imported through the PAT stream, auxin synthesized in the root tip, and local auxin transport, including the auxin lateral reflux. Lateral root development and emergence are controlled by auxin derived from the shoot for which the SL controlled PAT stream capacity and lateral auxin influx into the developing lateral root primordia (LRP) are the main determinants. Although in the flow diagram auxin is depicted as a positive regulator of root growth, auxin displays a dose-response curve with an optimum, such that supra-optimal auxin concentrations will have a negative effect (Ruyter-Spira et al., 2011). (2) The effect of SLs on RH elongation is dependent on both auxin and ethylene (ET) biosynthesis and signaling. It has been suggested that SLs negatively regulate auxin efflux (Koltai et al., 2010). If this would specifically occur in RH cells this would result in increased local auxin levels which stimulates RH elongation. This local action of SLs has not been proven yet. Alternatively, it may be that SLs affect auxin transport in the PAT stream and/or the root tip hereby indirectly affecting the auxin concentration in RH cells. ET acts downstream of SLs and has a direct effect on RH elongation but also interacts with the auxin pathway (Kapulnik et al., 2011b). Abbreviations: P, primordium; DM, distal meristem; PM, proximal meristem; AM, apical meristem; BM, basal meristem; TZ, transition zone; EZ, elongation zone; DZ, differentiation zone; FC, founder cell.

Auxin plays a crucial role in the regulation of bud outgrowth. Auxin is produced mostly in the shoot apex and young leaves (Ljung et al., 2001) and is transported basipetally toward the root apex in the stem through the polar auxin transport (PAT) stream (Petrasek and Friml, 2009) (Figures 2A–D). The PINFORMED (PIN) proteins, a family of plasma membrane auxin efflux carriers, determine the direction of this PAT stream. The PINs export auxin out of the cell across the cell membrane into the apoplast from where it is taken up by the next cell after which the whole process is repeated (Galweiler et al., 1998; Wisniewska et al., 2006).

Based on the pioneering work of Sachs (1968), one hypothesis concerning the regulation of bud outgrowth (canalization-based model) proposes that an initial auxin flux from an auxin source (shoot apex or buds) to an auxin sink (root) is gradually canalized into cell files with a large amount of PINs. These cell files will subsequently differentiate into vascular tissue through which auxin will be transported (Sachs, 1981; Domagalska and Leyser, 2011). Auxin export from buds is correlated with the initiation of bud outgrowth and therefore it is believed that buds need to export auxin in order to be activated [reviewed by Muller and Leyser (2011)]. In this model, all buds compete for the release of their auxin into the common main PAT stream in the stem. Auxin exported from active buds (auxin source) reduces the auxin sink strength of the PAT stream in the stem and inhibits other buds from auxin export into the PAT stream (Sachs, 1981; Domagalska and Leyser, 2011). In pea, it was indeed observed that active axillary buds of decapitated stems rapidly triggered PIN1 polarization thus enabling directional auxin export from the buds (Balla et al., 2011). Auxin application on the apex of the decapitated stem inhibited this PIN polarization and also prevented the canalization of laterally applied auxin (simulated as the secondary auxin source) (Balla et al., 2011).

SLs can inhibit shoot branching via its regulation on auxin transport. In Arabidopsis, *max* mutants (*max1*, *max2*, *max3*, *max4*) shown increased transcript levels of the *PIN1*/*3*/*4*/*6* genes and an increased auxin transport capacity in the primary stem when compared to wild type plants (Bennett et al., 2006). Treatment with N-1-naphthylphtalamic acid (NPA), an auxin transport inhibitor, led to a remarkable inhibition of bud outgrowth in *max* mutants in Arabidopsis and *dwarf* mutants in rice (Ishikawa et al., 2005; Bennett et al., 2006; Arite et al., 2007; Lin et al., 2009). Basal application of the synthetic SL GR24 reduced basipetal auxin transport and PIN1 accumulation in the plasma membrane of xylem parenchyma cells in wild type and biosynthetic *max* mutants but not in *max2* (Crawford et al., 2010). These results suggest that SLs dampen the PAT stream in a MAX2-dependent manner (Crawford et al., 2010).

To understand how SLs regulate auxin transport, Leyser's group performed a computer modeling study, in which different processes affecting PAT were simulated. The results from this study suggested that SLs may modulate PIN cycling between the plasma membrane and endosomes (Prusinkiewicz et al., 2009). More recent computer modeling work provided additional support for the canalization-based model for shoot branching control (Shinohara et al., 2013). In this study, the relationship between PIN1 accumulation, auxin transport and shoot branching was explored in three Arabidopsis mutants that show excessive shoot branching: *max2*, *gnom* (*gn*), and *transport inhibitor resistant3* (*tir3*) (Shinohara et al., 2013). Although all three mutants are highly branched, *max2* plants show high PIN:PIN1-GFP levels at the basal plasma membrane of stem parenchyma cells, accompanied by a high PAT capacity, while *tir3* and *gn* mutants show the opposite due to low PIN1 insertion rates at their plasma membranes (Shinohara et al., 2013). SL action was simulated to increase the PIN1 removal rate from the plasma membrane in these three excessive shoot branching mutants (Shinohara et al., 2013). Interestingly, the model predicted that, different concentrations of GR24 treatment can either inhibit or stimulate shoot branching, depending on the auxin transport status and concentration of the treated plant (Shinohara et al., 2013). This was confirmed to occur in *tir3*, in which a low concentration of GR24 promoted shoot branching (10 nM) while a higher GR24 concentration (0.1–1 μM) reduced branching (Shinohara et al., 2013). An explanation for this (maybe unexpected) induced shoot branching resulting from GR24 application is that, assuming that SLs systemically remove PIN1 from plasma membranes, auxin transport capacity is also systemically reduced. A slight reduction in auxin transport in tissue through which auxin is exported from the buds, would still allow bud outgrowth. However, due to this slight decrease, more buds can simultaneously participate in this auxin export process, hereby increasing the number of shoot branches that grow out. The above observation perfectly fits within the canalization theory for the regulation of shoot branching. Finally, the presumed SL mediated reduction in PIN1 endocytosis, used in the computer model, was finally experimentally confirmed and was shown to occur through a clathrin-dependent mechanism (Shinohara et al., 2013).

Consistent with the idea that SLs do not need to directly exert their branching-inhibiting function in the buds, *MAX2* in Arabidopsis is expressed throughout the plant, and particularly high in the vasculature of developing tissues (Stirnberg et al., 2007). Similarly, the other component involved in SL signaling, the α/β-fold hydrolase *D14*, is also expressed in vasculature tissues, especially in xylem parenchyma cells in leaves and stems in close vicinity to axillary buds (Arite et al., 2009). Taken together, depending on auxin transport status, SLs systemically regulate competition between buds to release their auxin into the stem, finally determining how many buds can be activated (Prusinkiewicz et al., 2009; Crawford et al., 2010; Shinohara et al., 2013).

An argument against the above described model is the fact that in Arabidopsis and pea, both wild type and SL biosynthetic mutants rapidly transport additional exogenously applied auxin, suggesting that their auxin transport capacity is not saturated (Brewer et al., 2009). In addition to this, another simulation study recently shown that the increase in auxin transport capacity in the main stem as a result of decapitation occurs too slow to explain the increased bud outgrowth (Renton et al., 2012). Rather, this simulation study suggested that if auxin canalization accounts for bud outgrowth, enhanced auxin levels in the bud itself may be the main driving force (Renton et al., 2012).

SLs as well as CKs are considered acropetally mobile signals that can enter the buds and directly regulate bud activity (second-messenger model) (Figure 2B). Controversial to the canalization-based model, this model emphasizes the local action of SLs. Expression patterns of SL biosynthetic genes reveal that SLs are likely synthesized in the vascular tissue of both roots and shoots. Root-derived SLs can be transported acropetally through the xylem sap stream (Kohlen et al., 2011). This is in accordance with grafting studies which already shown that branching-inhibitors can move from the roots to the shoot since the bushy phenotype of SL biosynthesis mutants can be rescued by grafting mutant shoots on wild type roots (Morris et al., 2001; Turnbull et al., 2002; Simons et al., 2007). However, grafting of wild type shoots on SL deficient mutant roots shown that this SL transport is not a prerequisite for branching inhibition, emphasizing the importance of local SL production in the stem. Besides, auxin upregulates the transcription of SL biosynthetic genes such as *CCD7* and *CCD8*, whereas decapitation results in decreased expression of these genes (Sorefan et al., 2003; Johnson et al., 2006; Arite et al., 2007; Brewer et al., 2009; Liang et al., 2010). According to Dun et al. (2013), the GR24 signal was profoundly perceived in the axillary buds rather than adjacent leaves in pea, supporting the direct local inhibitory effect of SLs in axillary buds. They also shown that the inhibitory effect of GR24 was not permanent, which is consistent with SLs' transient signaling role in mediating rapid plant developmental responses (Dun et al., 2013). The recently discovered SL transporter gene, petunia *PLEIOTROPIC DRUG RESISTANCE 1* (*PhPDR1)*, is particularly expressed in the vasculature and nodal tissues near the axillary buds (Kretzschmar et al., 2012), consistent with the fact that cellular transport of SLs is likely needed in this specific region. Indeed, shoot branching in the Petunia *pdr1* mutant is increased compared with the wild type, however not to the extent observed for SL biosynthetic mutants (Kretzschmar et al., 2012). This may point to a SL export-independent bud outgrowth inhibitory process. Considering the co-localization of the expression of *PIN1* and SL biosynthetic genes in vascular parenchyma cells, this SL export-independent process is potentially represented by the SL-mediated inhibition of the PAT capacity. Similar to SL, CKs are mostly synthesized in the roots, albeit with some biosynthesis also occurring in the shoot, and are also transported acropetally through the xylem (Chen et al., 1985; Nordstrom et al., 2004; Tanaka et al., 2006). In contrast to SLs, however, CKs promote bud outgrowth directly and auxin inhibits CK biosynthesis by suppressing the CK biosynthetic gene *IPT* (*ADENOSINE PHOSPHATE-ISOPENTENYL TRANSFERASE*) (Tanaka et al., 2006). Accordingly, decapitation or application of an auxin transport inhibitor led to enhanced expression of CK biosynthetic genes in nodal stem and increased CK levels in pea (Tanaka et al., 2006).

Consistent with the second-messenger model, SLs and CK, mediated by auxin, act antagonistically and locally in the buds to control bud outgrowth (Brewer et al., 2009; Ferguson and Beveridge, 2009; Dun et al., 2012). Based on decapitation and girdling experiments, it was hypothesized that growing axillary branches/buds affect auxin sink strength and also bud responsiveness to SLs (Ferguson and Beveridge, 2009). Auxin levels in the stem negatively regulate bud outgrowth by maintaining local high SL and low CK levels (Ferguson and Beveridge, 2009). Once buds are activated, auxin is exported into the stem to allow vasculature development (Ferguson and Beveridge, 2009). Recent research suggests that both SLs and CK can interact directly in buds to control bud outgrowth, converging at a common target in the bud, possibly a TCP transcription factor, BRANCHED1 (BRC1) (Dun et al., 2012). In eudicots such as Arabidopsis and pea, BRC1 has been suggested to be expressed in axillary buds and act downstream of SLs signaling during shoot branching inhibition (Aguilar-Martinez et al., 2007; Braun et al., 2012; Dun et al., 2012). The expression of the pea *PsBRC1* mostly occurred in the axillary buds and was up-regulated by application of GR24 and down-regulated by CK treatment (Braun et al., 2012; Dun et al., 2012). However, overexpression of *BRC1* ortholog *FC1* (*FINE CULM 1*) in rice could only partially rescue the tillering phenotype of the SL signaling mutant *d3* (Minakuchi et al., 2010). GR24 treatment did not significantly affect the expression of *FC1* whereas CK treatment did down-regulate its expression (Minakuchi et al., 2010). In maize, it seems that *BRC1* ortholog *TB1* (*TEOSINTE BRANCHED 1*) has evolved independent from SL signaling which may be explained by the fact that maize domestication is associated with a gain-of-function mutation in the *TB1* gene (Guan et al., 2012). Further research is still needed to clarify the regulatory mechanisms of the *BRC1* gene family and to find out whether additional factors in the axillary bud are involved in the regulation of bud outgrowth. Recent findings have shed some light on how other factors interact with FC1 in rice, targeting D14 to control shoot branching (Guo et al., 2013). Their results shown that OsMADS57, which is one of the transcription factors from the MADS-domain family, directly suppressed D14 transcription to control rice tillering, while FC1 could disturb this inhibitory effect of OsMADS57 on D14 by binding to the OsMADS57 (Guo et al., 2013).

Although second-messenger and canalization-based models look controversial, they can also be compatible since both local and systemic action of SL signaling are needed for adaptive plant responses. Figure 2 presents an overview of auxin, SLs and CK transport within the plant (left) and interactions between these hormones during the regulation of shoot and root development (right).

## Strigolactone interplay with other hormones in regulating root development

Plant root system displays a large plasticity which is required to guarantee resource acquisition in response to changing environments. Most dicot species have a typical allorhizic root system with a primary (tap) root (PR) and several orders of lateral roots (LR) (Osmont et al., 2007). Adventitious roots (AR) are initiated from non-root tissues such as the hypocotyl or stem. Most monocot species are characterized by a secondary homorhizic root system including the embryonic PR, post-embryonic shoot-borne crown roots, and LRs (Osmont et al., 2007). On a micro scale, the root system architecture also includes root hairs (RH) that expand the root surface area and hence the capacity of plants to withdraw nutrients and water from the soil (Gilroy and Jones, 2000).

### Primary root development

PR growth is mainly determined by the activity of the root apical meristem (RAM). This is a complex region of the root tip including a stem cell niche (SCN), a proximal meristem (PM), and a distal meristem (DM) (Figure 2D). Cell division, elongation, and differentiation in the RAM are tightly controlled by plant hormones. In this process, auxin is the main player. Different levels of cellular auxin have a different effect on gene expression, which determines cell fate. In roots, high auxin levels tend to stimulate cell division whereas lower levels favor cell expansion (Doerner, 2008). Auxin is mostly synthesized in the young leaves at the shoot apex (Ljung et al., 2001) and directionally transported through the vascular cambium of the shoot toward the RAM (Blilou et al., 2005; Petrasek and Friml, 2009). In roots, auxin is particularly accumulated in the quiescent center (QC), the columella initials and lateral root cap where auxin maxima are formed (Blilou et al., 2005; Petersson et al., 2009; Petrasek and Friml, 2009; Brunoud et al., 2012). Besides the auxin that is imported from the shoot, local auxin biosynthesis in the root also contributes to auxin homeostasis in the root tip (Chen and Xiong, 2009; Petersson et al., 2009). A major determinant of root growth is the auxin concentration gradient which is formed along the longitudinal axis of the root meristem. This concentration gradient is established due to the directional action of auxin transporters including auxin influx carriers such as AUXIN RESISTANT1(AUX1) and LIKE-AUX1 family and efflux carriers such as PINs and ATP-BINDING CASSETTE (ABC) transporters (Blilou et al., 2005; Kleine-Vehn et al., 2006; Grieneisen et al., 2007; Zazimalova et al., 2010). The directionality of the auxin flux is determined by the polar subcellular localization of these auxin efflux proteins (Sauer et al., 2006; Wisniewska et al., 2006; Petrasek and Friml, 2009). In the primary root, basally localized PIN1, PIN3, and PIN7 in the stele facilitate the acropetal auxin transport toward the root apex (Petrasek and Friml, 2009) (Figure 2D). In the columella, PIN3 and PIN7 redirect the auxin flow laterally toward the epidermis and the lateral root cap. PIN2 then facilitates the auxin flow from there upwards to the elongation zone (Petrasek and Friml, 2009). In addition, PIN2 in the cortex is also functional and fine-tunes both the rootward and shootward auxin flux, thus helps maintain auxin maxima at the root tip (Rahman et al., 2010). Finally, in the elongation zone, auxin is transported back into the main PAT stream through a lateral auxin reflux in the endodermis/cortex [as reviewed in Petrasek and Friml (2009)] (Figure 2D).

SLs are suggested to modulate the auxin gradient in the PR tip. The PR length of SL biosynthesis mutants (*max1*, *max3*, and *max4*) and SL signaling mutant (*max2*) is shorter than in wild-type plants (Ruyter-Spira et al., 2011). Application of GR24 (2.5 μM) rescued the short root phenotype of SL-deficient mutants but not of SL-insensitive mutant *max2* (Ruyter-Spira et al., 2011). The increased PR length was associated with an expansion of the meristem and transition zone sizes, through a higher number of smaller cells in both zones (Ruyter-Spira et al., 2011). Previously, modeling in which a reduction of the lateral auxin reflux was simulated shown a similar cellular patterning in the primary root tip (Grieneisen et al., 2007). This suggests that SLs may reduce the efficiency of the auxin lateral reflux into the main PAT stream which would affect auxin levels in both meristem and transition zones (Ruyter-Spira et al., 2011). Also consistent with these results, it has been demonstrated that expression of MAX2 under endodermis-specific SCARECROW (SCR) promoter in *max2* led to a wild-type level concerning meristem cell number, LR density, and RH elongation (Koren et al., 2013). Since PIN3-mediated auxin transport through the endodermis plays an important role in LR initiation (Marhavy et al., 2013), SLs' effects on PR growth and LR formation may indeed act through mediating auxin flux in the root tip (Koren et al., 2013). Interestingly, there was also evidence showing that SHORT HYPOCOTYL 2 (SHY2), which is the central mediator between auxin-CK antagonistic interaction in balancing cell differentiation with cell division in the meristem (Dello Ioio et al., 2008; Perilli et al., 2012), may be involved in endodermal SL signaling to regulate meristem size (Koren et al., 2013). Thus, SHY2 seems the converging point for auxin, CK as well as SLs. SLs may regulate PIN-based auxin flux via MAX2 and/or SHY2 (Koren et al., 2013); however, it is still not clear how SLs regulate SHY2. Besides, both *max2* and *shy2-31* mutants shown reduced sensitivity to CK treatment, suggesting that MAX2 and SHY2 participate in CK signaling in the root (Koren et al., 2013).

It has been suggested that the regulatory role of SLs in PR growth is mediated through their inhibitory effect on auxin-efflux carriers (Koltai et al., 2010; Ruyter-Spira et al., 2011; Koren et al., 2013). As mentioned in the previous part, SLs signaling has recently been found to rapidly trigger PIN1 depletion from plasma membrane of xylem parenchyma cells. However, compared to the shoot, the effect of SLs on PIN1 depletion in root is less drastic and less specific. No obvious short-term effect of GR24 on PIN1 accumulation was observed in the root tip even within 2 d (Shinohara et al., 2013). Only in the longer term (6 d), the inhibitory effect by GR24 treatment could be detected in the provascular region (Ruyter-Spira et al., 2011). This could be explained by SLs' feedback inhibition on auxin biosynthesis in young leaves and auxin transport capacity in the stem, which would lead to reduced auxin supply to the root (Ruyter-Spira et al., 2011). However, if the short term inhibitory effects of SLs on PINs are only expected to specifically occur in the endodermis cells of the transition zone (TZ), visualization of this process is technically challenging.

### Lateral root initiation and development

LR originates from a few auxin primed pericycle founder cells (FCs) located opposite of the xylem poles in the basal meristem (BM) of the parental root (Peret et al., 2009) (Figure 2D). LR formation is subsequently initiated through a series of anticlinal and periclinal cell divisions—controlled by auxin—in the primed FC. This process is promoted by the auxin reflux in the TZ (Casimiro et al., 2001; De Smet et al., 2007; Dubrovsky et al., 2008; Marhavy et al., 2013). Particularly, PIN3, which is transiently induced in the endodermis during early stages of LR initiation, enables proper auxin gradient for transition from FC to LR initiation (Marhavy et al., 2013). LR initiation is followed by tightly regulated cell divisions leading to subsequent LR primordial (LRP) development and finally LR emergence (Peret et al., 2009; De Smet, 2012) (Figure 2D). As LRP develop, auxin efflux carriers promote the accumulation of auxin in the tips of the multilayered LRP. The formation of a proper auxin maximum is a crucial event during LR development (Petrasek and Friml, 2009) (Figure 2D). The accumulated auxin in developing LR tips also serves as a local signal to remodel adjacent cells by inducing the expression of auxin influx carrier LAX3 (LIKE AUX1 3) in cortical and epidermal cells, which leads to cell separation in LRP overlaying tissues, thus enabling LR emergence (Swarup et al., 2008).

While LR initiation is dependent on auxin which is circling inside the root tip (and is derived from both the shoot and the root) (Reed et al., 1998; Casimiro et al., 2001; Marchant et al., 2002; Wu et al., 2007), subsequent LR development is solely sustained by shoot derived auxin transported to the parent root and into the LRP through the PAT stream (Casimiro et al., 2001; Bhalerao et al., 2002; Chhun et al., 2007; Wu et al., 2007). Inherent to these different auxin sources, the regulatory mechanisms controlling LR initiation and subsequent development are also different; however in both cases the control of PINs plays an important role.

SLs act as regulators for LR initiation and LRP development (Figure 2D). SL-deficient (*max3* and *max4*) and SL-insensitive (*max2*) mutants shown increased density of LRs compared with wild type (Kapulnik et al., 2011a). Treatment of Arabidopsis seedlings with increasing concentrations of GR24 shown that LR density is reduced when 2.5 μM GR24 is applied, however LR initiation is only reduced with 5 μM GR24 (Ruyter-Spira et al., 2011). Therefore it was concluded that the reduction in LR density observed with 2.5 μM GR24 results from a delay in LR development (Ruyter-Spira et al., 2011). Indeed, a LR developmental study shown a specific accumulation of LR stage V primordia according to the LR developmental scale of Malamy and Benfey (1997). The arrested primordia displayed reduced levels of auxin reporter DR5-GUS and pPIN1-PIN1-GFP, suggesting that reduced auxin levels inside LRP are responsible for their delayed development or arrest (Ruyter-Spira et al., 2011). Auxin is provided to the developing primordia by a PIN1-dependent auxin influx from the PAT stream in the stem into the LRP interior toward the LR cap. It has been shown that GR24 application to the roots of Arabidopsis reduced auxin levels in young leaves (Ruyter-Spira et al., 2011). Possibly, the SL-mediated reduction in auxin transport in the PAT stream temporarily increases auxin levels in vascular tissue throughout the plant, which negatively feeds back on auxin production in young leaves (or positively on auxin degradation), similar to what has been observed upon application of the auxin transport inhibitor NPA (Ljung et al., 2001). The role of SL signaling in lateral root development may also involve *SHY2* (Koren et al., 2013), which has been suggested to suppress LR initiation but promotes LR development by mediating PIN activity and auxin homeostasis (Goh et al., 2012). Endodermis-specific expression of *SCR:MAX2* in *max2* background restored LR density to a wild-type level. As PIN3-dependent auxin reflux between endodermis and pericycle has a critical function in LR initiation (Marhavy et al., 2013), the fact that MAX2-mediated endodermal SL signaling is sufficient to confer sensitivity to LR formation implies that SL signaling may regulate LR formation via modulating auxin flux in the elongation zone (Koren et al., 2013).

Hence the mechanism underlying the GR24 mediated reduction of LR initiation is likely similar to the one described above for PR growth, i.e., a reduction in auxin reflux through the transition zone. In addition, the above described reduction in shoot derived auxin likely also contributes to the reduction in both PR growth and LR initiation (Figure 2D).

### Root hair elongation

RHs are tip-growing, tube-like outgrowths that help to anchor roots in the soil and assist in the uptake of nutrients and water (Gilroy and Jones, 2000). In the differentiation zone (DZ) of the root, RH emerge at the base of the epidermis cells. RH development can be divided into two stages: determination of hair/non-hair cells and hair morphogenesis (Lee and Cho, 2008). A cell in contact with two cortex cells will develop into a hair cell. RH initiation has been suggested to be directly mediated by optimal auxin levels and signaling, whereas ET's effect is indirect and likely to act through regulating intracellular auxin levels (Muday et al., 2012). RH elongation requires an optimal intracellular auxin level which is regulated by auxin efflux and influx carriers. Auxin efflux PIN2 facilitates auxin supply through basipetal auxin transport from the root apex to the RH differentiation zone (Cho et al., 2007). PIN2 in the cortex has recently been shown to fine-tune both the rootward and shootward auxin flux (Rahman et al., 2010). Modeling of the auxin flow suggests that auxin influx carrier AUX1-dependent transport through non-hair cells can maintain auxin supply for developing hair cells and sustain RH outgrowth (Jones et al., 2009). ET also plays a positive role in regulating RH elongation (Tanimoto et al., 1995; Rahman et al., 2002). Both the Arabidopsis *ein2* (*ethylene insensitive 2*) mutant and ET-resistant mutant *aux1* exhibited decreased RH length (Rahman et al., 2002). Application of a low concentration of 1-naphthaleneacetic acid (NAA) (10 nM) could restore RH length of ET-resistant mutant *aux1* (Rahman et al., 2002). However, a much higher level of NAA (100 nM) was needed to recover RH length of *ein2* to the wild-type level, suggesting that the loss of ET signaling makes roots less sensitive to auxin (Rahman et al., 2002). SLs interact with auxin and ET in regulating RH elongation (Figure 2D). In tomato, a high dose of exogenous GR24 (27 μM) resulted in shorter and fewer RH than in the control (Koltai et al., 2010). The authors suggested that the effect of SLs is mediated via an effect on auxin efflux carriers (Koltai et al., 2010). In *Arabidopsis*, treatment with a low dose of GR24 increases the RH length in WT and in *max3* and *max4* mutants but not in *max2*, indicating the positive regulatory role of SLs in RH elongation, mediated via the MAX2 protein (Kapulnik et al., 2011b). Concerning RH elongation, SL signaling mutant *max2* has a similar sensitivity to ET precursor ACC as wild type, whereas ET signaling mutants *ein2-1* and *etr1-1*(*ethylene resistant1-1*) show reduced sensitivity to GR24, suggesting that SL signaling is not necessary for the ET response but ET signaling is involved in the SL response (Kapulnik et al., 2011b). Furthermore, SL application stimulates expression of ET biosynthetic genes (Kapulnik et al., 2011b). Taking together, these results suggest that ET biosynthesis is necessary for SLs to have an effect on RH elongation and that ET acts downstream of SLs (Figure 2D). The relationship between SLs and auxin in RH formation was also explored by the same authors. RH elongation upon IAA application in *max2* was similar to that of wild type, suggesting that SL signaling is not necessary for the auxin response. In contrast, auxin perception mutant *tir1-1* exhibited a reduced response to GR24 compared with the wild type, implying that auxin perception is needed for the SL response (Kapulnik et al., 2011b). However, the reduced sensitivity of *tir1-1* to GR24 may also be due to its reduced response to ET since *tir1-1* also shows reduced sensitivity to ACC. Moreover, the double mutant *aux1-7ein2-1* (insensitive to auxin and ET) shows reduced sensitivity to GR24 compared with the wild type upon RH elongation. Therefore, the effect of SLs on RH elongation is dependent on both auxin and ET biosynthesis and signaling while ET signaling also directly interacts with the auxin pathway (Kapulnik et al., 2011b) (Figure 2D).

As mentioned above, RH initiation and elongation takes place in epidermis cells (Lee and Cho, 2008). Endodermal SL signaling, mediated by MAX2, is still sufficient to confer sensitivity for RH elongation, suggesting the effect of SLs on RH elongation is likely to occur in a non-cell-autonomous manner (Koren et al., 2013).

### Adventitious root formation

ARs are post-embryonic roots that arise from non-root tissues. They can be induced by direct organogenesis from differentiated cells or from callus formed upon mechanical damage such as a cutting (Li et al., 2009). The formation of ARs in tomato occurs in the lower part of the hypocotyl as well as from the shoot-root junction. IAA application enhances AR formation in tomato hypocotyls in a dose-dependent manner (Negi et al., 2010). In rice calli, overexpression of auxin biosynthetic gene *YUCCA1* (*YUC1*), results in increased numbers of ARs (crown roots) as well as active crown root formation in the elongated node of the stem, suggesting that increased auxin production promotes AR development from both callus and stem (Yamamoto et al., 2007). Interestingly, in the stem, *OsYUC1-GUS* is expressed in the parenchyma cells surrounding the vascular bundles, suggesting local auxin biosynthesis in the vasculature of the stem (Yamamoto et al., 2007). In addition, AR emergence and development in rice are significantly suppressed in *OsPIN1* RNAi lines (Xu et al., 2005), suggesting an essential role of PIN1-dependent PAT during the process of AR initiation and development. Since SLs have been found to trigger PIN1 depletion from xylem parenchyma cells in the stem (Shinohara et al., 2013), it is also plausible to predict their inhibitory effect on PAT and thus AR development.

Indeed, studies on Arabidopsis and pea (*Pisum sativum*) show that SLs negatively regulate AR formation (Rasmussen et al., 2012a,b). SL biosynthetic and signaling mutants of both species displayed increased number of AR compared with wild type. It was suggested that SLs suppress AR formation by inhibiting the very early divisions of FCs (Rasmussen et al., 2012b). When *MAX2* is expressed in *max2* under the control of a xylem-specific promoter *NST3* (*NAC SECONDARY WALL THICKENING PROMOTING FACTOR3*), the AR formation is restored to the wild type level. This is consistent with the fact that *MAX2* is expressed in vasculature tissues throughout the plant. The authors suggest that SL signaling in the xylem is sufficient to mediate the formation of pericycle-derived AR. Interestingly, etiolation is known to induce AR formation in hypocotyls and this process is stimulated in all *max* mutants. The expression of *MAX3* and *MAX4* in wild type hypocotyls is induced upon light exposure, suggesting that local SL biosynthesis is involved in the regulation of AR formation during the process of de-etiolation (Rasmussen et al., 2012b). SL treatment of Arabidopsis wild type and *max* biosynthesis mutants (but not the signaling mutant *max2)*, results in a reduction in AR number even in the presence of elevated auxin levels (such as in *35S: YUC1* plants). The auxin response mutant *auxin resistant 1 (axr1)* and the *axr1max1-4* double mutants hardly form ARs. Auxin application (although not all concentrations) increases the number of ARs in *max* mutants (Rasmussen et al., 2012b). These findings indicate that SLs can at least partially revert the positive effect of auxin on AR formation and *AXR1* functions upstream of SLs in the early stages of AR initiation (Rasmussen et al., 2012b). The authors also investigated possible crosstalk between SLs and CK in regulating AR development as CK are known to suppress AR formation. CK responsiveness is not impaired in the SL mutants and CK mutants are also SL-responsive, indicating that SLs and CK act independently in AR formation (Rasmussen et al., 2012b).

## SL and auxin action during secondary growth

Plant growth initiated by apical meristems leads to development of primary tissues such as epidermis, vascular bundles and leaves. In addition to primary growth, plants, especially tree species, also display secondary growth during which they expand their growth axes laterally. Secondary growth depends on the activity of the vascular cambium which originates from the procambium and parenchyma cells (Ye, 2002). The vascular cambium has the capacity to divide and form a continuous ring of meristem cells located between the primary xylem and the phloem in the vascular bundles (Ursache et al., 2013). The cylindrical layer of cambium undergoes cell division, resulting in new xylem on the inside and new phloem on the outside (Ye et al., 2002; Ursache et al., 2013). There is strong evidence that procambium patterning is regulated by PIN1-dependent PAT (Scarpella et al., 2004, 2006). Also secondary xylem differentiation was shown to be associated with reduced PAT. The Arabidopsis *interfascicular fiber* mutant (*ifl1*) displays reduced secondary growth (Zhong and Ye, 2001). The authors shown that reduced expression of auxin efflux carriers and the resulting reduced PAT along the inflorescence stems and hypocotyls in this mutant lead to a block of vascular cambium activity (Zhong and Ye, 2001).

SLs have recently been proven to positively regulate secondary growth (Figure 2C). SL biosynthetic and signaling mutants all displayed reduced cambium activity compared with wild type. Local application of GR24 stimulates cell division in the interfascicular cambium in wild type and all Arabidopsis SL biosynthetic *max* mutants and to a lesser extent in the *max2* signaling mutant (Agusti et al., 2011). Remarkably, the *max2* mutant is still slightly responsive to GR24 which is not consistent with its complete insensitivity in other processes such as shoot branching and root development. This suggests that there may also be other factors involved in the transduction of the SL signal in this particular physiological process (Agusti et al., 2011). In this study of Agusti et al. (2011), shoot branching is not affected by GR24 application showing that the effect of SLs on cambium development in inflorescence stems is mechanistically independent from the effect they have on shoot branching (Agusti et al., 2011). Interestingly, although the *max1* mutant displays reduced secondary growth, its auxin concentration, signaling and transport are enhanced. This suggests that the effect of SLs on secondary growth is direct and independent of auxin accumulation (Agusti et al., 2011). In addition to this, local NPA application, which reduces the initially enhanced auxin transport capacity observed in the *max* mutants, does not restore secondary growth, suggesting that SL biosynthesis and signaling are required for auxin to stimulate cambium activity. This conclusion is supported by the fact that GR24 application to the auxin insensitive *axr1-3* mutant results in a similar increase in cambial activity as observed for wild type and the *max* mutants. Collectively, these results suggest that SLs function downstream of auxin in the regulatory pathway of secondary growth in Arabidopsis (Agusti et al., 2011). However, the observed remaining cambium activity in *max1* cannot be ignored. It would suggest that either auxin also has a direct effect or that residual SLs are still present in the *max1* mutant background.

## SL and other hormones during seeds germination

SLs have been identified as germination stimulants for seeds of parasitic plants *Orobanche spp*. and *Striga spp*. These parasitic plants seeds are usually dormant in soil and germinated only when they are close to host roots. Previous studies shown that ABA levels decrease during seeds pre-conditioning of *O. minor* (Chae et al., 2004). Still, seed dormancy release depends on an additional reduction of ABA levels which was recently shown to be mediated through ABA catabolism which is triggered by GR24 application (Lechat et al., 2012). Other hormones such as CK and ET can promote parasitic plant seeds germination in the absence of SLs (Logan and Stewart, 1991; Babiker et al., 1993, 1994; Sugimoto et al., 2003), suggesting that they may act downstream of SLs; whereas CK promotes germination by enhancing ET biosynthesis (Babiker et al., 1993). Furthermore, GA is necessary but not sufficient to trigger *Striga* seeds germination (Toh et al., 2012).

Currently, model plant Arabidopsis is also being used to explore hormone interactions, including SLs, during seed germination. Based on thermoinhibition experiments, a positive role of SLs in Arabidopsis seeds germination was revealed (Toh et al., 2012). Both SLs biosynthetic and signaling mutants shown enhanced sensitivity to high temperature which is a constraint for normal germination (Toh et al., 2012). GR24 could not only alleviate thermoinhibition by decreasing ABA levels and increasing GA levels, but also break secondary dormancy in Arabidopsis. Nice comparisons were made between hormone interactions occurring during the alleviation of thermoinhibition in parasitic and non-parasitic seeds germination (Toh et al., 2012). In both cases, SLs reduce the ABA:GA ratio, leading to enhanced germination activity. To trigger Striga seed germination, SLs also positively regulate CK which contributes to ET production (not proven for Arabidopsis yet) (Toh et al., 2012). However, as expected when considering the difference in germination behavior between parasitic plants and Arabidopsis, differences between hormone signaling networks were also reported. GA, for instance, is sufficient to counteract thermoinhibition in Arabidopsis seeds but is not sufficient to do so in parasitic plants seeds (Chae et al., 2004; Toh et al., 2012). Besides, parasitic plants seeds are very sensitive to SLs that are exuded from host plants, suggesting their evolutionary dependence on hormone interaction (Toh et al., 2012). Light signaling related topics concerning seeds germination will be discussed in the following The Response to Light section. Interestingly, a smoke-derived compound, karrikin, has similar effects on seed germination in a MAX2-dependent manner (Nelson et al., 2011). The *kai2* (karrikin insensitive 2) mutant seeds are insensitive to GR24. It was suggested that there is a butenolide-based signaling mechanism via KAI2 which is distinct from SL signaling, providing an adaptive response to smoke (Waters et al., 2012b).

## Hormone interactions in response to environmental stimuli

Plants, unlike animals, are sessile organisms and hence require phenotypic plasticity, which is the ability of a certain genotype to produce different phenotypes in response to varying environmental conditions (Pfennig et al., 2010). Meristem development is of vital importance for the adaptation of plants to changes in the environment. Regulation of axillary meristem outgrowth, for example, is one of the major strategies that plants adopt to adjust their body plan, leading to changes in shoot branching. Another mechanism to modify the body plan is to alter secondary growth of stems and roots by regulating development of lateral meristem tissue, especially the vascular cambium (Agusti and Greb, 2013), allowing plants to regulate root and shoot thickness. Collectively, all plant meristems are closely coordinated to face environmental challenges during plant development. In the following paragraphs we will elaborate on how SLs and other plant hormones are involved in the regulation of two different environmentally regulated physiological processes, the response to light and the response to nutrient shortage.

### The response to light

Light is a highly variable environmental factor affecting plant growth and development. Changes in light quality and intensity affect multiple processes in plants, such as intensively studied shade avoidance syndrome (SAS). During this response, plants are able to detect a decrease in the R:FR and initiate morphological changes that help plants to compete with their neighbors (Franklin, 2008), such as elongation of internodes, hypocotyls, and petioles, reduced shoot branching and leaf development, inhibited root growth, early flowering, and reduced seed set in the long term (Ruberti et al., 2012). The stimulation of the elongation responses can be as rapid as a few minutes and the process is reversible. The photoreceptors responsible for the response to changes in light quality in the red and far-red regions are the phytochromes, including PhyA to PhyE in higher plants.

Light also affects the levels of plant hormones and in turn, plant hormones affect the photoreceptor signal transduction (Wang et al., 2013). Shade has been reported to induce a rapid increase in auxin levels, its PIN-based transport (i.e., PIN1 and PIN3) and auxin signaling, resulting in enhanced elongation growth (Tao et al., 2008; Keuskamp et al., 2010; Hornitschek et al., 2012). Notably, it has been shown that *PIN1* expression was regulated by the photomorphogenesis repressor COP1 (CONSTITUTIVE PHOTOMORPHOGENIC 1), which is suppressed by light-activated PHYB. COP1 not only controlled the transcription of *PIN1* and the capacity of the PAT stream in the hypocotyls but also affected PIN1 and PIN2 intracellular distribution in the root tip thus affecting root elongation. This suggests that COP1 efficiently coordinates both root and shoot growth under changing light conditions (Sassi et al., 2012).

SLs were shown to be essential components of the low R:FR mediated reduction of bud outgrowth. In Arabidopsis it was shown that both *BRC1* and the SL biosynthetic and downstream signaling genes *MAX4* and *MAX2* were needed to suppress branching during low R:FR conditions (Finlayson et al., 2010). In addition to this, functional *AXR1*, was also essential for the control of shoot branching under low R:FR conditions, confirming that auxin signaling is important during shade avoidance reactions (Tao et al., 2008) and is probably needed to induce SL biosynthesis. Indeed, auxin was shown to induce SL biosynthetic gene expression under normal light condition (Hayward et al., 2009). It's very likely that it's the similar case under shade: auxin levels and PAT stream are promoted under shade, which may enhance SL biosynthesis, leading to reduced bud outgrowth.

A low R:FR and/or inactive PHYB also induce an elongation response in branches. Interestingly, the Arabidopsis *max2* mutation inhibited the elongation response of rosette branches in the presence of the *phyB* mutation, while *axr1-12* and *max4* maintained the elongation response of branches in the *phyB* mutant (Finlayson et al., 2010). Also for other light regulated plant growth characteristics, such as decreased hypocotyl growth and de-etiolation, MAX2 dependency has been observed while the SL biosynthetic mutants did not display the corresponding photomorphogenic phenotypes. For instance, while *max2* is hyposensitive to red, far-red, and blue light, leading to longer hypocotyls (Stirnberg et al., 2002; Shen et al., 2007; Nelson et al., 2011), this was not the case for *max1*, *max3*, and *max4* (Shen et al., 2012). Therefore, it was suggested that MAX2 regulates photomorphogenesis in a SL-independent manner, and may form complexes consisting of different ligands and/or substrates. In this respect it is intriguing that not only the response to SLs, but also to smoke derived compounds called karrikins, requires MAX2 (Nelson et al., 2011). An alternative explanation could be that the SL biosynthetic mutants tested in these studies are leaky, and still produce sufficient SLs to result in different phenotypes when compared to the signaling mutant. Based on altered expression patterns of GA and ABA biosynthesis and catabolic genes in Arabidopsis *max2* seeds, in combination with a *max2* specific germination phenotype, it was hypothesized that MAX2 would also affect photomorphogenesis by modulating hormonal levels in a non-SL dependent manner (Shen et al., 2012). However, again, it could be that the hormonal levels in the SL biosynthetic mutants are not enough reduced to result in a phenotype. It would therefore be interesting to include SL biosynthetic double or triple mutants in these experiments. A direct link between SLs and photomorphogenesis has been suggested (Tsuchiya et al., 2010). It was shown that SLs inhibit hypocotyl elongation in the dark. However, it must be noted that non-physiological levels of GR24 (50 μM) were applied. A mechanistic explanation for the MAX2/SL role in photomorphogenesis was provided with the discovery that GR24 (10 μM) mediates nuclear exclusion of COP1, which leads to the stabilization of HY5 (ELONGATED HYPOCOTYL 5) and reduced hypocotyl elongation (Tsuchiya et al., 2010). This led to the intriguing conclusion that SL application can mimic light under dark conditions (Tsuchiya et al., 2010). However, in contrast to above results (Tsuchiya et al., 2010), it was recently found that HY5 is not necessarily required for MAX2-dependent SL regulation of hypocotyl growth (Waters and Smith, 2013). It was proposed that HY5 and MAX2 act in separate signaling pathways during early light-mediated seedling development and that they may subsequently interact, in later developmental stages, downstream of auxin and light signaling (Waters and Smith, 2013).

### The response to nutrient deprivation

Nutrient deprivation is another important abiotic stress frequently encountered by plants. Phosphorus (P), for example, is one of the essential macronutrients required by plants but only the inorganic phosphate (Pi) is the phosphorus form which is accessible for plants. As roots are the main site for Pi acquisition, plant roots usually cope with Pi-limiting conditions by investing more energy into root growth, resulting in reduced shoot/root ratio (including inhibited shoot branching), inhibited PR elongation and enhanced LR and RH growth (Williamson et al., 2001; Linkohr et al., 2002; Niu et al., 2012). It has been shown that the root tip is involved in sensing low Pi (Svistoonoff et al., 2007).

In Arabidopsis, the *phosphorus starvation-insensitive* (*psi*) mutant, displaying reduced inhibition of PR growth and reduced LR and RH growth under Pi-limited conditions, shown less sensitivity to auxin and enhanced ability to sustain auxin response in the root tip than wild type plants under low Pi, suggesting that low Pi can increase the sensitivity of roots to auxin (Wang et al., 2010). The enhanced auxin sensitivity induced by Pi deprivation is conferred by an increased expression of *TIR1*, which accelerates the degradation of AUX/IAA proteins (Perez-Torres et al., 2008).

In addition to auxin, SLs are also important regulators of root architecture under Pi-limiting conditions. SL production in roots is promoted by Pi starvation (Yoneyama et al., 2007; Lopez-Raez et al., 2008; Jamil et al., 2011). Interestingly, while LR development in Arabidopsis SL biosynthetic and signaling mutants was increased during normal Pi conditions, LR outgrowth was decreased during Pi starvation (Ruyter-Spira et al., 2011). Similarly, in rice, crown root elongation in wild type was increased in Pi-deficient media while *d10* and *d14* mutant plants did not show such response (Arite et al., 2012). Particularly the results in Arabidopsis suggest that the increase in SL production under Pi-limited conditions is necessary for the expansion of the root system, allowing the plant to explore a larger area of the soil for nutrients. That this is due to an interaction with auxin is suggested by the results of an experiment in which GR24 was applied to Arabidopsis plants growing on medium also containing auxin (NAA) which resulted in a more rapid elongation of lateral roots than in the absence of GR24 (Ruyter-Spira et al., 2011). Moreover, GR24 application to plants grown with sufficient Pi caused a more severe reduction in lateral root number compared with plants grown under Pi starvation (Ruyter-Spira et al., 2011). Because Pi starvation increases auxin sensitivity (Perez-Torres et al., 2008; Koltai, 2012) and GR24 application was shown to decrease auxin levels in the leaves, it is likely that the final effect of GR24 (or SLs in general) in the low Pi response depends on the auxin status of the plant, as affected by the environment (Pi level) of the plant.

The effect of SL on Pi starvation-mediated changes in RH density also sheds light on the mechanism by which SL affect auxin signaling. Arabidopsis SL biosynthetic and signaling mutants shown a remarkably lower RH density, than wild type plants and only the response of the SL biosynthetic mutant *max4*, not that of *max2*, could be rescued by exogenous treatment with GR24 (Koltai, 2012; Mayzlish-Gati et al., 2012). These results could be explained by the absence of low Pi mediated induction of TIR1 in *max2* while TIR1 expression is induced in wild type plants. This would render SL mutant plants less sensitive to auxin during Pi starvation. Moreover, this SL-mediated RH response to low Pi was suggested to be independent or downstream of the ET signaling pathway, while only auxin, and not ET was able to restore the relatively low RH density in the *max2* mutant (Koltai, 2012; Mayzlish-Gati et al., 2012).

The expression of SL exporter *PDR1* is also induced by Pi deprivation. PDR1 is localized in the plasma membrane of sub-epidermal cells of roots, facilitating SL exudation into the rhizosphere and promotes the symbiotic interaction with AM fungi and hence Pi uptake by the plant (Kretzschmar et al., 2012). SL production in the root is relatively high. A part of this SL pool is transported upwards to the shoot. It has been shown in Arabidopsis and tomato that under low Pi, increased levels of SLs travel through the xylem (Kohlen et al., 2011). This systemic mode of action allows SLs to rapidly regulate aboveground architecture by altering PIN accumulation (Shinohara et al., 2013), thus facilitates nutrient re-allocation. However, under Pi deficiency, transcript levels of SL biosynthetic genes were also slightly increased in the shoot (Umehara et al., 2010), suggesting that local SL biosynthesis in the shoot also contributes to the branching inhibition observed during low Pi conditions. However, currently it is not known to what extent this local production is sufficient, and if it is, why SLs are transported to the shoot through the xylem. One explanation could be that long-distance transport of SLs provides a feedback mechanism for auxin levels (through production and/or degradation) in auxin producing tissues in the shoot, as was demonstrated to occur upon GR24 application in Arabidopsis seedlings (Ruyter-Spira et al., 2011). In conclusion, SLs play multiple roles in the response of plants to low Pi conditions. They not only improve Pi acquisition by improving AM fungi symbiosis but also act as long-distance signal to optimize shoot architecture in a nutrient-limited environment and regulate root architecture in such a way that Pi uptake can be improved.

In summary, plants have evolved multiple adaptive mechanisms to achieve phenotypic plasticity, not only by regulating whole plant architecture, but also by balancing nutrient allocation among different organs in response to changing environments. Plant hormones play a crucial role in these adaptive responses and their intricate interaction enables fine-tuned responses to many different changes in the environment.

## Perspective

Plants exhibit a high degree of plasticity, which is defined by their ability to adjust their development to changes in the environment. Hormone interactions can fine-tune the plant response and determine plant architecture when plants are challenged by environmental stimuli such as nutrient deprivation and canopy shade. One of the essential nutrients plants strongly respond to is phosphate. Modern agriculture is highly dependent on its application, and its finite resource is worrying and deserves immediate attention. Future strategies need to focus on lower phosphate fertilizer application accompanied by improved phosphate use efficiency (PUE) by agricultural crops. Improved PUE is a highly desirable trait to which also root architecture contributes. Since SLs are involved in different plant developmental processes leading to plant architectural changes, including root architecture, more knowledge about their role, particularly under phosphate limiting conditions, is highly desirable. This includes the low phosphate mediated regulation of SL transport within the plant and the exudation to the rhizosphere as well as the local regulation of SL biosynthesis and transport in close vicinity to the buds.

SL crosstalk with other plant hormones is still a research area in its infancy, certainly at the cellular and genetic level. As we have pointed out in this review, a common target for many plant hormones is the regulation of auxin levels and gradients through their effect on PINs. The exact mechanism of how SLs do this however still needs to be resolved. Because different hormonal and environmental signals also interact with each other this is very complex. Computational modeling and simulations may facilitate the interpretation of complicated datasets, leading to predictions or the establishment of new models.

Finally, the intriguing structural diversity in SLs observed in plants and its relevance for differential regulation of various plant developmental processes is of great interest. Improved knowledge about SL perception and downstream signaling mechanisms will shed more light on the biological relevance of this structural diversity. The discovery of genetic variation and favorable alleles of genes involved in SL diversification and downstream signaling processes would be an interesting asset to future breeding programs as it will help to fine-tune SL action in such a way that maximum benefit is obtained in agriculture (improved PUE, better crop architecture, etc.), without negative side effects (germination of parasitic weeds).

### Conflict of interest statement

The authors declare that the research was conducted in the absence of any commercial or financial relationships that could be construed as a potential conflict of interest.

## Abbreviations

P: primordium
SAM: shoot apical meristem
DM: distal meristem
PM: proximal meristem
AM: apical meristem
BM: basal meristem
TZ: transition zone
EZ: elongation zone
DZ: differentiation zone
FC: founder cell
RAM: root apical meristem
PR: primary root
RH: root hairs
LR: lateral root
LRP: lateral root primordia
SL: strigolactone
CK: cytokinin
ET: ethylene
PAT: polar auxin transport.
